# Commentary: Reimagining Nursing Education in Ireland Through New National Standards

**DOI:** 10.1111/jan.70415

**Published:** 2025-11-28

**Authors:** John Gilmore, Freda Browne

**Affiliations:** ^1^ School of Nursing, Midwifery and Health Systems University College Dublin Dublin Ireland

In 2022 a full review of the Irish undergraduate nursing and midwifery curricula was carried out, and subsequently, following a consultation the Nursing and Midwifery Board of Ireland (NMBI) introduced new standards for nursing registration programmes (NMBI 2025). This represents a profound reorientation of the principles underpinning nursing education in Ireland. These standards move decisively towards an outcomes‐based model, situating the social determinants of health as a central framework for understanding health, illness, and disability. In doing so, they signal a deliberate pivot in how the profession is prepared for practice in a rapidly changing health system, while maintaining continuity with European Union directives that guarantee quality, safety, and professional mobility across member states.

Ireland's 2025 nursing education reforms mark a decisive shift in how nurses are prepared for practice. By centring the social determinants of health and rebalancing clinical learning towards primary and community care, the new standards position nursing as a socially engaged, equity‐focused profession. While these reforms are nationally significant, they also speak to wider international debates on competence‐based education, health equity, and the future of nursing.

The historical trajectory of nursing education in Ireland provides an important backdrop. For most of the twentieth century, nurses were trained through hospital‐based apprenticeship systems. While these models produced generations of clinically skilled practitioners, they tended to limit exposure to wider social, policy, and research contexts. The Commission on Nursing (1997) catalysed a decisive break with that tradition, recommending that nursing preparation be relocated to higher education. By 2002, all pre‐registration programmes had been transferred to universities and institutes of technology establishing degree‐level preparation as the national standard (Morris et al. 2019). For more than two decades, every graduate nurse in Ireland has therefore completed an honours degree, embedding nursing firmly within the academy and strengthening its evidence base.

The new standards represent the next stage of this evolution. Where the reforms of the early 2000s secured the academic legitimacy of nursing, the 2025 reforms explicitly reframe education around equity, inclusion, and community health. By placing the social determinants of health at the centre of curricula, the NMBI acknowledges that nurses must be able to respond not only to clinical presentations but also to the wider conditions in which people are born, live, and age. In practice this means preparing nurses to understand and respond to issues such as poverty, housing insecurity, racism, and social exclusion. It also means equipping them to work in partnership with individuals and communities to promote wellbeing, reduce disparities, and advocate for health justice (Abu and Moorley 2023). In taking this stance, Ireland positions itself at the forefront of European and international efforts to reorient nursing education towards social accountability and health equity, aligning with global priorities set out by the World Health Organization (WHO) and the International Council of Nurses (ICN).

This reorientation aligns nursing education with broader health system reform. Sláintecare, Ireland's national strategy for universal, community‐oriented healthcare, seeks to shift the centre of gravity of the health system away from acute hospitals and towards integrated primary and community services. Nursing education has historically remained disproportionately anchored in hospital settings, even after its transition to universities. The new standards deliberately broaden practice learning environments, with greater emphasis on placements in primary care, general practice, and community‐based services. In doing so, they prepare nursing graduates for the reality that the majority of health needs are met outside hospital walls, and that future nursing careers will increasingly involve working in multidisciplinary teams embedded in communities.

This national agenda reflects a wider international call. Since the late 1990s, the WHO has urged Member States to rebalance health systems by shifting resources and expertise from hospitals to primary and community settings. Central to this vision is the recognition that nurses are critical to delivering integrated, home‐ and community‐based care, and that their education must extend beyond hospital traditions to prepare them for more independent and autonomous practice (WHO 2021).

Crucially, these developments are not undertaken in isolation. The standards retain alignment with Directive 2005/36/EC of the European Parliament, which prescribes minimum requirements for nursing education across the EU, including the 4600 h of combined theory and practice and the balance between clinical and academic instruction. This alignment ensures that Irish graduates continue to benefit from automatic recognition across the European Union, while at the same time allowing the NMBI to set national priorities. The dual emphasis on international comparability and local responsiveness reflects an astute balancing of obligations and opportunities.

The shift from prescriptive to outcomes‐based standards represents another significant philosophical change. Previous models tended to specify detailed lists of curricular content and clinical experiences. While this ensured consistency, it risked narrowing innovation and constraining flexibility. By contrast, the 2025 standards articulate broad outcomes, creating space for higher education institutions to design curricula that respond to contemporary challenges, local contexts, and diverse student needs (NMBI 2025). This approach is consistent with wider trends in health professions education internationally, where competence‐based frameworks have been adopted to ensure graduates are not only knowledgeable but adaptable and capable of critical reflection (Zumstein‐Shaha and Grace 2023).

Internationally, few nursing education systems have gone this far. While the WHO and ICN call for socially accountable health professional education, implementation remains patchy. Ireland therefore offers a case study of what it looks like to embed these principles at the level of regulatory standards. The question, however, is whether this bold ambition can be matched with the necessary resources, placements, and faculty development to make it real in classrooms and communities.

Flexibility in curriculum design also creates opportunities for pedagogical innovation. The standards explicitly encourage inclusive, learner‐centred approaches, and offer scope to embed Universal Design for Learning (UDL) principles across teaching, assessment, and practice learning. UDL requires educators to design from the outset for variability, offering multiple means of engagement, representation, and expression. In practice, this ensures that students with different learning styles, abilities, and needs can access and succeed in programmes without compromising professional standards (Gilmore et al. 2022). Alongside UDL, learner‐centred pedagogies emphasise dialogue, reflection, and co‐production, positioning students as active agents in their own education. Such approaches are particularly resonant in nursing, a discipline that values partnership, relational practice, and responsiveness to context.

On the other hand, outcomes‐based models carry risks. Without clear guidance and investment, variability between programmes may widen, potentially undermining comparability of graduates. It is likely that success will depend less on the regulatory shift itself and more on how higher education institutions and practice partners interpret and implement it.

There are also workforce implications arising from these new standards. Ireland faces well‐documented nursing shortages, with demand projected to rise as demographic change and chronic illness place pressure on services, particularly in community settings (Connolly and Flanagan 2024). Recruiting and retaining nursing students is therefore a matter of strategic importance (WHO 2021). Educational models that are inclusive, flexible, and engaging have the potential to enhance student satisfaction and retention (Gilmore et al. 2022). By preparing graduates for the realities of community, primary and integrated care, these standards may also support a more balanced distribution of the workforce, reducing the historical over‐reliance on hospital roles and ensuring nursing capacity aligns with health system reform.

Beyond workforce numbers, however, lies a broader question of professional identity. The 2002 reforms embedded nursing as a degree‐based academic discipline. The 2025 reforms now extend that trajectory by affirming nursing as a socially engaged and equity‐oriented profession. By integrating social determinants, community practice, and inclusive pedagogies into core expectations, the standards invite educators and students alike to conceive of nursing as not only clinical but also civic, not only scientific but also relational, and not only responsive but also preventive.

The challenge ahead is implementation. The adoption of standards alone does not guarantee transformation. Realisation will depend on universities, practice partners, and policymakers embracing the opportunities offered. This includes investing in high‐quality community placements, ensuring academic staff are supported to adopt inclusive pedagogies, and fostering co‐design of curricula with service users and communities. Confirmation on the structures to support students beyond hospital settings, where an established infrastructure of clinical placement co‐ordinators, practice development leads and placement allocation leads, is awaited and likely to be a challenge for smaller community healthcare settings. The radical shift in approach to curriculum development also requires robust evaluation to assess whether new approaches deliver on their promise of preparing nurses who are not only competent but also critically engaged and equity‐focused (Abu and Moorley 2023).

More than two decades of degree‐level education have provided Irish nursing with a strong academic foundation. The adoption of new standards in 2025 builds on that foundation with a clear vision for the future. By balancing European alignment with national priorities, by shifting from prescription to outcomes, and by centring the social determinants of health, the standards mark a significant moment in the evolution of nursing education. If embraced fully, they have the potential to prepare a generation of nurses who are clinically expert, socially responsive, and ready to contribute to an equitable health system.

For the global nursing community, the Irish case offers both inspiration and caution. It demonstrates how regulatory standards can reorient education towards health equity, but also highlights that such reforms demand structural investment and critical evaluation. If realised fully, they could prepare a generation of nurses who are not only clinically expert but also socially responsive, a model from which many health systems could learn.

## Funding

The authors have nothing to report.

## Conflicts of Interest

The authors declare no conflicts of interest.

## Data Availability

No data were generated or utilised for this commentary, and no fieldwork permissions were required.
